# Rice Stress Associated Protein 1 (OsSAP1) Interacts with Aminotransferase (OsAMTR1) and Pathogenesis-Related 1a Protein (OsSCP) and Regulates Abiotic Stress Responses

**DOI:** 10.3389/fpls.2016.01057

**Published:** 2016-07-19

**Authors:** Kamakshi S. Kothari, Prasant K. Dansana, Jitender Giri, Akhilesh K. Tyagi

**Affiliations:** ^1^National Institute of Plant Genome Research, New DelhiIndia; ^2^Department of Plant Molecular Biology, University of Delhi South Campus, New DelhiIndia

**Keywords:** abiotic stress, *Arabidopsis*, A20/AN1, protein interactions, zinc-finger

## Abstract

Stress associated proteins (SAPs) are the A20/AN1 zinc-finger containing proteins which can regulate the stress signaling in plants. The rice SAP protein, OsSAP1 has been shown to confer abiotic stress tolerance to plants, when overexpressed, by modulating the expression of endogenous stress-related genes. To further understand the mechanism of OsSAP1-mediated stress signaling, OsSAP1 interacting proteins were identified using yeast two-hybrid analysis. Two novel proteins, aminotransferase (OsAMTR1) and a SCP/TAPS or pathogenesis-related 1 class of protein (OsSCP) were found to interact with OsSAP1. The genes encoding OsAMTR1 and OsSCP were stress-responsive and showed higher expression upon abiotic stress treatments. The role of *OsAMTR1* and *OsSCP* under stress was analyzed by overexpressing them constitutively in *Arabidopsis* and responses of transgenic plants were assessed under salt and water-deficit stress. The *OsAMTR1* and *OsSCP* overexpressing plants showed higher seed germination, root growth and fresh weight than wild-type plants under stress conditions. Overexpression of *OsAMTR1* and *OsSCP* affected the expression of many known stress-responsive genes which were not affected by the overexpression of *OsSAP1*. Moreover, the transcript levels of *OsSCP* and *OsAMTR1* were also unaffected by the overexpression of *OsSAP1*. Hence, it was concluded that OsSAP1 regulates the stress responsive signaling by interacting with these proteins which further regulate the downstream stress responsive gene expression.

## Introduction

Plants are constantly challenged by a number of environmental extremities, like water-deficit, temperature, salinity, and pests during their life. To withstand these adversities, they have developed specialized mechanisms which lead to the modulation of their gene expression patterns for adaptive development and growth. Different stress-responsive genes are induced and repressed to play crucial roles in stress signaling. The overall stress signaling is a complex network of different pathways that ultimately confers the appropriate response depending on the type of stress encountered. To understand these signaling mechanisms, both forward and reverse genetics approaches have been employed. Through these approaches, many transcription factors and regulatory proteins have been identified which, if modulated, can affect the whole signal transduction pathway (Bhatnagar-Mathur et al., 2008; Hirayama and Shinozaki, 2010). The regulatory proteins often interlink and control multiple stress signaling pathways and hence can function as the master regulators of stress response (Chinnusamy et al., 2004). Zinc-finger proteins (ZFPs) are a group of regulatory proteins which have diverse functions in a variety of organisms (Takatsuji, 1999). They can function as transcription factors, RNA binding proteins and protein modification enzymes to regulate the growth, development and stress responses of an organism (Eulgem et al., 2000; Englbrecht et al., 2004; Agarwal et al., 2007; Moreno-Risueno et al., 2007).

Stress associated proteins (SAPs) are a class of ZFPs composed of two zinc-finger domains, an N-terminal A20 domain and a C-terminal AN1 domain. These proteins have recently been identified as novel stress regulatory proteins in plants (Giri et al., 2013). These types of proteins have also been identified in animals as regulators of immune signaling and many other responses including biotic/abiotic stresses (Heyninck and Beyaert, 2005). *SAP* gene family is prevalent in many organisms including plants, animals, protists, and fungi. Majority of SAPs have been found to be stress-inducible and some of the members from different plants have been characterized to confer abiotic stress tolerance in transgenic plants (Giri et al., 2013). In contrast, OsSAP7 has recently been characterized to be a negative regulator of ABA responsive stress signaling (Sharma et al., 2015). Similarly, ZFP185 (OsSAP4) has also been found to be involved in GA and ABA signaling and negatively regulates abiotic stress responses (Zhang et al., 2015). The role of SAPs in regulation of biotic stress responses is also emerging. Banana SAP gene, *MusaSAP1* overexpressing transgenic plants showed strong up-regulation of polyphenol oxidase (PPO) encoding transcripts which are well-known to play a role in biotic defense pathway (Sreedharan et al., 2012). A recent study has revealed a role of *OsSAP1* in regulating basal defense against pathogen infection via up-regulation of known defense-responsive genes such as *PR* genes (Tyagi et al., 2014). SAPs are considered to be regulatory proteins and it has been suggested that they can affect the stress signaling by interacting and modulating the activity of target proteins, though their molecular functions are poorly known. The SAPs have been identified as novel E3 ubiquitin ligases in analogy to their animal counterparts. *Arabidopsis* SAP5, ubiquitinates AtMBP1, a negative regulator of ABA and stress signaling, and targets it for degradation (Kang et al., 2011, 2013). Similarly, OsSAP7 has also been shown to possess E3 ligase activity (Sharma et al., 2015). In addition, SAPs can function as redox sensor as shown for AtSAP12, which can change its oligomeric conformation depending upon the cellular redox potential (Stroher et al., 2009). Besides, SAPs can homo-/hetero-dimerize and interact with other proteins via their zinc-finger domains (Kanneganti and Gupta, 2008; Giri et al., 2011). OsSAP1 and OsSAP11 have been found to interact with a receptor-like cytoplasmic kinase, OsRLCK253, which itself is stress-responsive and its overexpression in *Arabidopsis* conferred tolerance to abiotic stresses. It was speculated that either the kinase can activate the SAPs through phosphorylation or SAP proteins can regulate the activity of RLCK253 (Giri et al., 2011). In a similar way, it is expected that these proteins can interact with many other proteins and involve in different functions, which needs to be elucidated.

In this study, an attempt has been made to identify the proteins interacting with OsSAP1 using yeast two-hybrid assay and the involvement of interacting proteins in stress response was evaluated by gene overexpression in *Arabidopsis*.

## Materials and Methods

### Plant Material, Growth Conditions and Stress Treatments

Rice (*Oryza sativa* ssp. *indica*) cv. Pusa Basmati-1 was used for preparation of cDNA library and expression analysis in stress conditions. Surface-sterilized seeds were grown in hydroponics in culture room at 28 ± 2°C with 16 h/8 h light/dark cycle and 100–125 μmol m^-2^ sec^-1^ fluence for 7 days. For expression studies, 7-day-old seedlings were given different stress treatments for 3, 8, and 24 h, and unstressed seedlings in RO water were kept as control. For salt and oxidative stress treatments, seedlings were incubated in 100 mM NaCl and 10 μM methyl viologen (MV) solutions, respectively. For water-deficit stress, seedlings were kept on three layers of dry tissue paper in culture room for the indicated time points.

*Arabidopsis thaliana* (ecotype Col-0) was used for generation of transgenic plants and as wild-type control in transgenic analysis as well as expression analysis of target genes. *Arabidopsis* plants were grown in culture room maintained at 22 ± 1°C with continuous illumination (100 μmol m^-2^ sec^-1^). Ten-day-old seedlings were harvested for gene expression analysis.

### Yeast Two-Hybrid Library Screening

The cDNA for yeast two-hybrid screening was prepared from 7-day-old rice seedlings treated with 3 h of water-deficit stress using Matchmaker^TM^ Library Construction & Screening Kit (Clontech, USA) as per the manufacturer’s instructions and was transformed along with pGADT7-Rec vector in yeast AH109 cells to generate the prey library [activation domain (AD) fusion library]. The library was screened with SAP1- binding domain (BD) as bait and the transformants were selected on SD/-His/-Leu/-Trp media supplemented with 2.5 mM 3-AT. The putative positive clones were identified by DNA sequencing. To reconfirm the protein interactions, the positive AD clones (*AMTR1* and *SCP*) were amplified and cloned in pGADT7-Rec vector and co-transformed with the BD clones (SAP1-BD, A20-BD, AN1-BD, SAP1ΔA20ΔAN1-BD) followed by selection on SD/-His/-Leu/-Trp and SD/-Ade/-His/-Leu/-Trp. The interactions were further confirmed by the activation of a third reporter gene *LacZ* by quantitative β-galactosidase assay using *o*-nitrophenyl-β-D-galactopyranoside (ONPG) as a substrate following the instructions in Yeast Protocol Handbook (Clontech, USA).

### Gene Cloning

The cDNA was prepared from total RNA extracted from 7-day-old rice seedlings using RevertAid H Minus First Strand cDNA Synthesis Kit (Thermo Scientific, USA) as per manufacturer’s protocol. The coding sequences of *OsAMTR1* and *OsSCP* were amplified from cDNA using gene-specific primers (Supplementary Table S1) and were cloned in desired vectors. For yeast two-hybrid study, the above sequences were cloned in pGADT7-Rec vector, while OsSAP1 (1–495 bp), A20 domain (10–171 bp), AN1 domain (250–493 bp) and the linker region between A20 and AN1 domain (SAP1ΔA20ΔAN1-BD; 180–305 bp) encoding sequences were cloned in pGBKT7 vectors. To analyze the protein-protein interactions using BiFC (Bimolecular Fluorescence Complementation), the coding sequences of *OsSAP1* and the interacting protein genes, i.e., *OsAMTR1* and *OsSCP*, were cloned in pSPYCE(MR) and pSPYNE(R)173 vector, respectively (Waadt et al., 2008) to generate a fusion with the C- and N-terminal fragment of eYFP. For subcellular localization, *OsAMTR1* and *OsSCP* were cloned in frame with the coding sequence of YFP in pSITE3CA vector (Chakrabarty et al., 2007) using Gateway^®^ technology. Similarly, for overexpression studies, *OsAMTR1* and *OsSCP* were cloned in binary vector pMDC32 using Gateway^®^ cloning strategy under the control of dual *CaMV35S* promoter.

### Generation of *Arabidopsis* Transgenic Plants

The *35S*::*OsAMTR1* and *35S*::*OsSCP* constructs were transformed in *Agrobacterium* strain GV3101 and transformed in *Arabidopsis* plants using floral dip method (Clough and Bent, 1998). The transgene segregation was assessed using Chi-square test to identify the lines with single T-DNA insertion events. The expression of transgene in the homozygous T3 lines was analyzed using qRT-PCR and three lines of each transgenic plant (line # 11, 17, and 23 of *35S*::*OsAMTR1*; line # 4, 22, and 27 of *35S*::*OsSCP*), having single insertion as determined by segregation analysis in T1 generation, were used for further analyses.

### Stress Treatment and Assessment of Effects on Plant Growth

To assess the effect of abiotic stress conditions on germination efficiency of *35S*::*OsAMTR1* and *35S*::*OsSCP Arabidopsis* plants, surface-sterilized seeds of WT and transgenic lines were germinated on MS medium plates supplemented with different concentrations of NaCl (100–150 mM) and mannitol (250–400 mM) for salt stress and water-deficit stress, respectively. As a control, seeds were also germinated on MS basal plates. After stratification at 4°C, plates were incubated in continuous light (100 μmol m^-2^ sec^-1^) at 22 ± 1°C and per cent seed germination was scored every day for 4 days. The emergence of radicle was considered as an indication of seed germination.

To analyze the seedling growth under stress, the WT and transgenic seeds were germinated on MS plates supplemented with 100 mM NaCl or 200 mM mannitol. After stratification, the plates were incubated in continuous light (100 μmol m^-2^ sec^-1^) at 22 ± 1°C in vertical position to allow root growth. After 10 days, root length and fresh weight of seedlings were recorded.

### Subcellular Localization of Fusion Proteins

The *35S*::*YFP-OsAMTR1* and *35S*::*YFP-OsSCP* constructs were transiently transformed in onion epidermal cells using particle bombardment (Bio-Rad, USA). The cells were analyzed under TCS-SP2 Confocal Laser Scanning Microscope (Leica, Germany) for YFP fluorescence after 18–20 h of incubation in dark at room temperature. To plasmolyse the epidermal cells, the onion peels were incubated in 5% NaCl (w/v) solution for 2–3 min before visualizing under microscope.

### Protein Interaction Using BiFC (Bimolecular Fluorescence Complementation) Approach

For BiFC assay, the protocol described by Waadt and Kudla (2008) was used. In brief, the fusion construct *35S*::*YFP_C155_*-*OsSAP1*, *35S*::*YFP_N173_*-*OsAMTR1* and *35S*::*YFP_N173_*-*OsSCP* were transformed in *Agrobacterium* strain GV3101. The transformed strains at an OD_600_ of 0.5 were mixed with *Agrobacterium* p19 strain (suppressor of silencing) at an OD_600_ of 0.3. The mixture was infiltrated in 4–5 weeks old *Nicotiana benthamiana* leaves and observed for YFP fluorescence (488 nm excitation laser) 5–6 days after infiltration under TCS SP5 confocal microscope (Leica, Germany).

### Gene Expression Analysis

The expression levels of transgene and target genes in *Arabidopsis* and/or rice plants were analyzed by qRT-PCR. For expression analysis of transgene, leaf tissues from mature plants were harvested, while for stress marker genes, 10-day-old seedlings of *Arabidopsis* transgenic and WT (Col-0) plants were harvested. For expression analysis in stress condition, the 7-day-old rice seedlings were used for stress treatments and RNA extraction, as described above. The cDNA was prepared from 1 μg of total RNA using High-Capacity cDNA Reverse Transcription Kit (Applied Biosystems, USA). The primers for qRT-PCR were designed by Primer Express (version 3.0) software (Supplementary Table S2). Quantification of amplified product was done by Fast SYBR^®^ Green Master Mix (Applied Biosystems, USA) and relative expression levels were analyzed by comparative Ct (2^-ΔΔCt^) method.

## Results

### Identification of OsSAP1 Interacting Proteins

**FIGURE 1:**
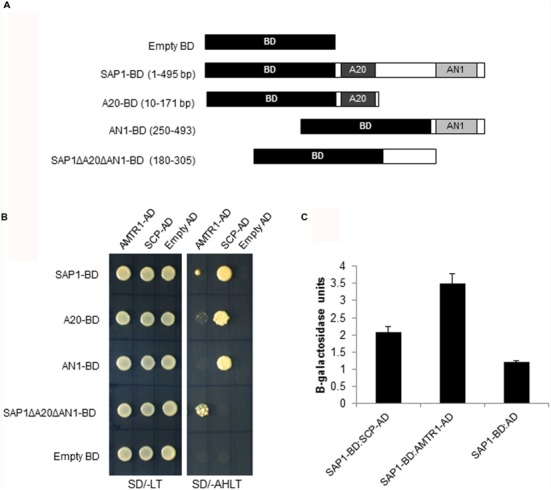
**Identification of proteins interacting with OsSAP1. (A)** The DNA binding domain (BD) was fused to different deletions of OsSAP1 to prepare bait constructs. **(B)** Yeast cells co-transformed with bait constructs shown in **(A)** and AD clones (SCP-AD, and AMTR1-AD) were plated on SD/-Leu/-Trp (SD/-LT) and SD/-Ade/-His/-Leu/-Trp (SD/-AHLT) media to test the interaction. Empty BD vector was co-transformed with empty AD clones to serve as negative control. **(C)** Quantification of β-galactosidase activity in the transformed yeast cells using *o*-nitrophenyl-β-galactopyranoside (ONPG) as a substrate. Negative controls: Empty AD transformed with SAP1:BD. The histogram represents mean ± SE of three biological replicates.

OsSAP1 has been shown to be involved in abiotic stress response (Mukhopadhyay et al., 2004; Giri et al., 2013; Dansana et al., 2014). Its interacting proteins under stress were identified using the yeast two-hybrid library screening approach (Clontech, USA). The cDNAs generated from water-deficit stress treated seedlings were fused with GAL4 AD to generate the prey library. This library was screened using OsSAP1, fused with the GAL4 BD, as bait (**Figure 1A**). After initial screening of the putative positive colonies by sequencing and removal of false positives, nine prey clones were found to have inserts in frame with AD domain (Supplementary Figure S1). For the next round of screening, the prey clones were amplified (full length) and cloned in fusion with AD followed by two-hybrid analysis with OsSAP1-BD. To further determine the region of OsSAP1 that can specifically interact with the AD clones, different deletions of OsSAP1, consisting of A20, AN1 and the linker region between A20 and AN1 domain (SAP1ΔA20ΔAN1), were fused with BD domain (**Figure 1A**) and co-transformed with the AD clones followed by selection on SD/-Ade/-His/-Leu/-Trp media (AHLT). Out of nine clones, two were found to interact with full length OsSAP1 and its domains. These were identified as novel uncharacterised genes namely, *OsAMTR1* (LOC_Os05g39770) and *OsSCP* (LOC_Os07g03710) (**Figure 1B**). OsAMTR1 was predicted to be a member of Aminotransferase class-III family of proteins. The 391 amino acid long OsAMTR1 protein carries a pyridoxal phosphate dependent transferase domain lying between 3 and 389 amino acid residues (Supplementary Figure S2). OsSCP was found to be a member of ‘SCP/TAPS (Sperm Coating Protein/Tpx-1/Ag5/PR-1/Sc7)’ protein family (Mizuki and Kasahara, 1992; Cantacessi et al., 2009). The coding sequence of *OsSCP* was found to be 507 nucleotides long encoding a protein of 168 amino acids (Supplementary Figure S3). The OsSCP protein contains a CAP (Cysteine-rich secretory proteins, Antigen 5, Pathogenesis-related 1 proteins; InterProScan: IPR014044) domain between 11 and 168 amino acid residues. The literature survey identified *OsSCP* to encode a pathogenesis-related 1a (OsPR-1a) protein (Agrawal et al., 2000). OsSCP was found to interact strongly and with equal efficiency with both A20 and AN1 domains of OsSAP1, whereas, OsAMTR1 interacted more strongly with linker region between A20 and AN1 domains (SAP1ΔA20ΔAN1). The interactions of these two proteins were further confirmed by the activation of LacZ reporter using quantitative β-galactosidase assay (**Figure 1C**; Supplementary Figure S4).

### Subcellular Localization of OsAMTR1 and OsSCP

**FIGURE 2:**
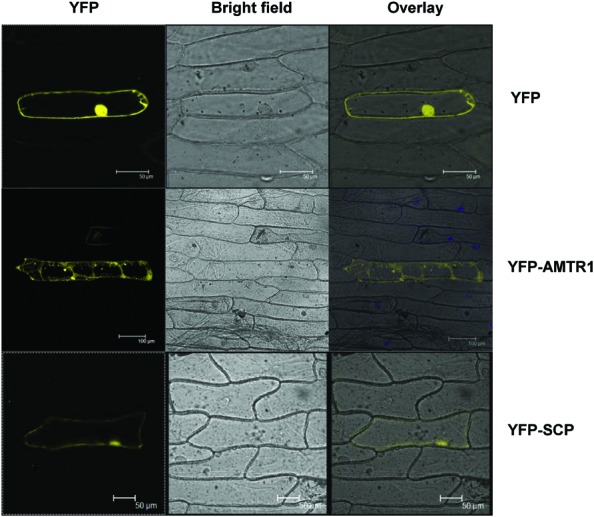
**Subcellular localization of OsAMTR1 and OsSCP.** The YFP fusion constructs (YFP-AMTR1 and YFP-SCP) were transformed transiently in onion epidermal cells and were visualized by confocal microscope. Empty vector (YFP) was used as a negative control.

**FIGURE 3:**
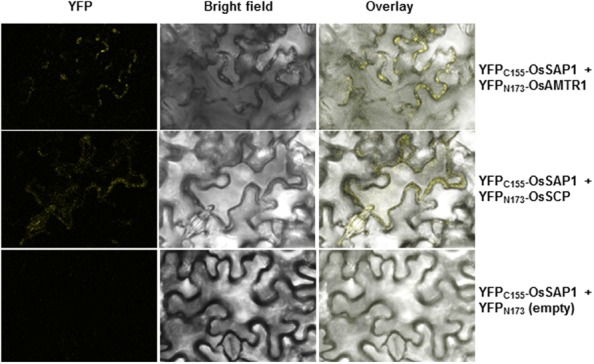
**Confirmation of interaction of OsSAP1 with OsAMTR1 and OsSCP using BiFC approach.** *OsSAP1* was cloned in pSPYCE(MR) while *OsAMTR1* and *OsSCP* were cloned in pSPYNE(R)173 vector to generate the fusions with C- and N-terminus of YFP, respectively. *35S*::*YFP_C155_-OsSAP1* was co-infiltrated separately with *35S*::*YFP_N173_-OsAMTR1* and *35S*::*YFP_N173_-OsSCP* in *Nicotiana benthamiana* leaves and visualized under confocal microscope for the YFP fluorescence. As a negative control, *35S*::*YFP_C155_-OsSAP1* was co-transformed with empty SPYNE(R)173.

To delineate their cellular functions, the coding regions of these genes were fused to the C-terminal of YFP fluorescent marker gene and subcellular localization was studied. The YFP-OsAMTR1 fusion protein was found to be distributed in whole cytoplasm including cytoplasmic strands and some dotted structures, although no signal was observed in the nucleus of onion epidermal cells. However, YFP-OsSCP was localized in cytoplasm as well as nucleus, evenly (**Figure 2**). Further, being a secretory protein, OsSCP was expected to localize in extracellular space, However, YFP-OsSCP remained in cytoplasm as revealed in plasmolysed cells (Supplementary Figure S5). The interaction of OsSAP1 with OsAMTR1 and OsSCP was analyzed *in vivo* using BiFC approach. OsSAP1 was fused with C-terminal half of YFP protein while OsAMTR1 and OsSCP were fused with N-terminal half of YFP and co-infiltrated in *N. benthamiana* leaves to analyze the YFP fluorescence using confocal microscope. The complex of YFP_C155_-OsSAP1 and YFP_N173_-OsAMTR1 showed YFP signal in whole cell. Similarly, YFP_C155_-OsSAP1/YFP_N173_-OsSCP complex also showed signal in tobacco cell (**Figure 3**). This confirmed the interactions of OsSAP1 with OsAMTR1 and OsSCP.

### The Genes Encoding OsSAP1 Interacting Proteins Were Stress-Inducible

**FIGURE 4:**
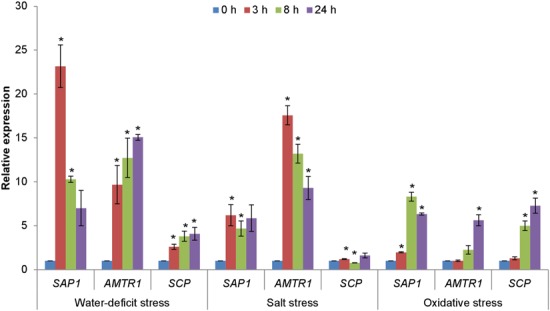
**Expression analysis of genes encoding interacting proteins of OsSAP1 under different abiotic stress treatments.** Seven-day-old seedlings were treated with NaCl (200 mM), water-deficit (air drying), and methyl viologen (MV; 10 μM) induced oxidative stress for different time durations (0, 3, 8, and 24 h) and expression levels were quantified by qRT-PCR. The histogram represents the mean ± SE of three biological replicates. Asterisks represent significant difference with respect to unstressed (0 h) condition as determined by *t*-test at *p*-value <0.05.

The expression patterns of *OsAMTR1* and *OsSCP* in different abiotic stress conditions was analyzed by treating the 7-day-old rice seedlings with salt, water-deficit and oxidative stress for different time durations and expression analysis was done using qRT-PCR (**Figure 4**). Under water-deficit treatment, very high induction (~23-folds) was seen for *OsSAP1* at 3 h, which is consistent with its early induction under water-deficit condition as reported earlier (Mukhopadhyay et al., 2004; Dansana et al., 2014). The *OsSAP1* expression decreases at later time-points. At 3 h, *OsAMTR1* showed about 10-fold induction and the expression further increased with time. Similar pattern of expression was observed for *OsSCP*, with 2.5–4 folds upregulation. Under salt stress, the highest expression was seen in *OsAMTR1*, which gets induced to a very high level (~17-folds) at 3 h and the expression decreases, thereafter. The expression of *OsSAP1* was about five folds higher than control and was almost constant at all time-points. But, weak induction was observed for *OsSCP*. All the genes showed up-regulation ≥2 folds under oxidative stress and expression increased with time except in *OsSAP1* where after 8 h, expression level decreased slightly. These expression profiles imply that although expression patterns of genes encoding OsSAP1 interacting proteins are not exactly similar to that of *OsSAP1*, all the genes are responsive to abiotic stress conditions.

### Evaluation of Response of *Arabidopsis* Transgenic Plants Overexpressing *OsAMTR1* and *OsSCP* during Stress Condition

To functionally characterize the *OsAMTR1* and *OsSCP* under abiotic stress, these genes were overexpressed in *Arabidopsis* under the control of *CaMV35S* promoter. After confirming the transgenic nature by PCR, the expression of transgene was analyzed using qRT-PCR. The transgenic plants showed no visible phenotypic differences with the wild-type plants under normal growth conditions (Supplementary Figures S6 and S7). These lines also showed similar seed germination rate as compared to wild-type under normal growth conditions (Supplementary Figure S8). Three independent lines from each transgenic (AT-11, AT-17, and AT-23 of *35S*::*OsAMTR1*; SCP-4, SCP-22, and SCP-27 of *35S*::*OsSCP*) were selected for further stress analysis.

#### Functional Characterization of *OsAMTR1* under Salt and Water-Deficit Stress

**FIGURE 5:**
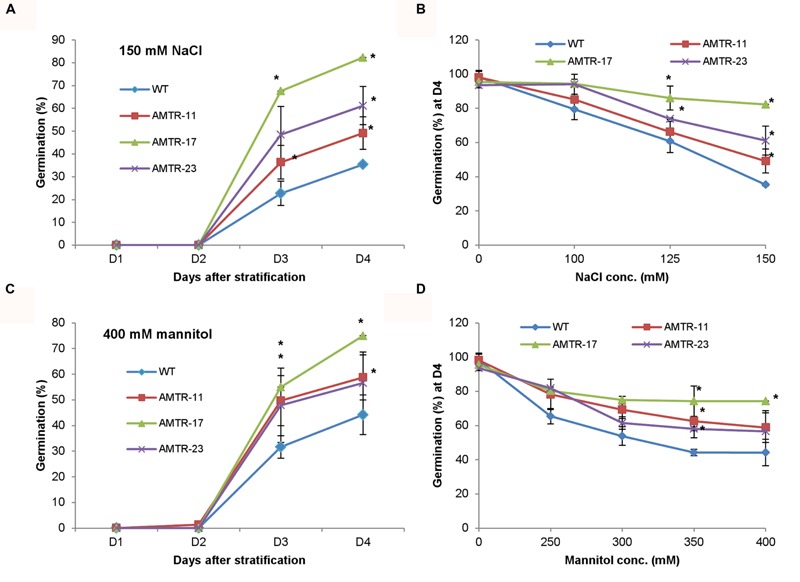
**Effect of abiotic stress on seed germination efficiency of *35S*::*OsAMTR1 Arabidopsis* plants.** Wild-type (WT) and transgenic (AMTR-11, AMTR-17, and AMTR-23) seeds were plated on MS medium supplemented with **(A)** 150 mM NaCl and **(C)** 400 mM mannitol and per cent seed germination was scored for 4 days after stratification. **(B,D)** The per cent seed germination at day 4 after stratification on variable concentrations of NaCl and mannitol, respectively. The graphs represent the mean ± SE of three biological replicates (*n* = 50). Asterisks represent significant difference with respect to wild-type as determined by *t*-test at *p*-value <0.05.

**FIGURE 6:**
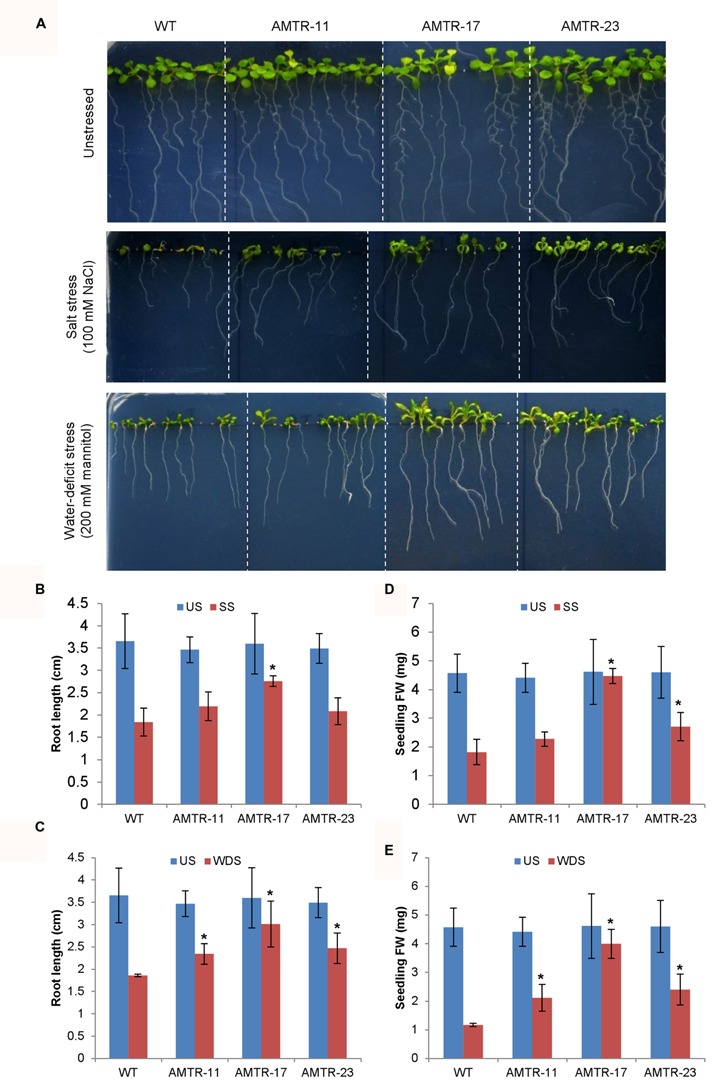
**Effect of abiotic stress on root growth and biomass accumulation of *35S*::*OsAMTR1 Arabidopsis* plants.** Seeds of WT and transgenic lines (AMTR11, AMTR17, and AMTR23) were plated on MS medium supplemented with 100 mM NaCl and 200 mM mannitol and plates were incubated in vertical position. **(A)** Morphology of WT and transgenic lines, **(B,D)** root length and **(C,E)** fresh weight (FW) of individual plant after 10 days of growth. The histograms represent mean ± SE from three biological replicates (*n* = 10). Asterisks represent significant difference with respect to wild-type as determined by *t*-test at *p*-value <0.05. SS, salt stress, US, unstressed; WDS, water-deficit stress.

The effect of overexpression of *OsAMTR1* on plant during abiotic stress conditions was analyzed by treating the plants with different stresses and evaluation of response of transgenics in comparison to wild-type. To assess the effect of salt and water-deficit stress on the seed germination efficiency, wild-type and *35S*::*OsAMTR1* transgenic seeds were germinated on MS medium supplemented with different concentrations of NaCl or mannitol and per cent seed germination were scored for 4 days. The transgenic seeds were found to have higher germination efficiency as compared to wild-type seeds under both high salt and mannitol conditions (**Figures 5A,C**). Even at lower concentrations of NaCl and mannitol, the *35S*::*OsAMTR1* plants showed higher germination than wild-type (**Figures 5B,D**). Similarly, to study the effect of salt and water-deficit stress during seedling growth stage, the root growth and biomass accumulation in 10-day-old seedlings of wild-type and *35S*::*OsAMTR1* transgenic plants were analyzed. The seeds of wild-type and transgenic lines were plated on salt and mannitol containing media and after 10 days of growth, transgenic plants were found to have longer roots than WT plants (**Figures 6A–C**). Further, the fresh weight of transgenic plants was higher than the wild-type plants (**Figures 6D,E**). The improvement in root growth and biomass accumulation was more prominent under water-deficit as compared to salt stress.

#### Functional Characterization of *OsSCP* under Salt and Water-Deficit Stress

**FIGURE 7:**
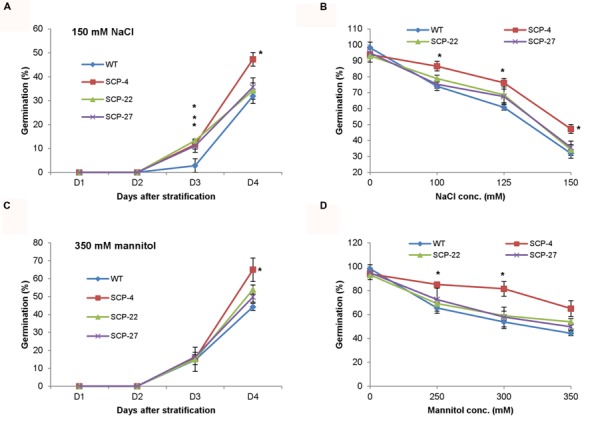
**Effect of abiotic stress on seed germination efficiency of *35S*::*OsSCP Arabidopsis* plants.** Wild-type and transgenic (SCP-4, SCP-22, and SCP-27) seeds were plated on MS medium supplemented with **(A)** 150 mM NaCl and **(C)** 350 mM mannitol and per cent seed germination was scored for 4 days after stratification. **(B,D)** The per cent seed germination at day 4 of stratification on variable concentrations of NaCl and mannitol, respectively. The graphs represent mean ± SE from three biological replicates (*n* = 50). Asterisks represent significant difference with respect to wild-type as determined by *t*-test at *p*-value <0.05.

**FIGURE 8:**
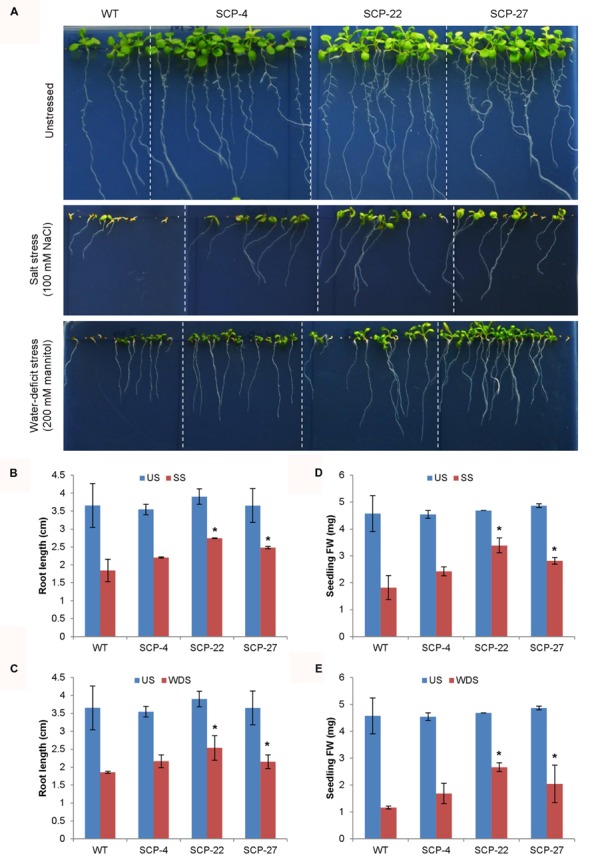
**Effect of abiotic stress on root growth and biomass accumulation of *35S*::*OsSCP Arabidopsis* plants.** Seeds of WT and transgenic lines (SCP-4, SCP-22, and SCP-27) were plated on MS medium supplemented with 100 mM NaCl and 200 mM mannitol and plates were incubated in vertical position. **(A)** Morphology of WT and transgenic lines, **(B,D)** root length and **(C,E)** FW of individual plant after 10 days of growth. The histograms represent mean ± SE from three biological replicates (*n* = 10). Asterisks represent significant difference with respect to wild-type as determined by *t*-test at *p*-value<0.05. SS, salt stress, US, unstressed; WDS, water-deficit stress.

As stated earlier, *OsSCP* was overexpressed in *Arabidopsis* under the control of *CaMV35S* promoter and *35S*::*OsSCP* transgenic plants were assessed for their response under abiotic stress conditions. Transgenic seeds showed higher germination efficiency than wild-type only at high concentration of NaCl (150 mM), while, at lower concentrations, transgenic seeds had slightly higher or almost similar germination efficiency as that of wild-type (**Figures 7A,B**). Similarly, at different concentrations of mannitol, only marginally higher germination was observed in transgenic seeds than wild-type, except for line SCP-4, which showed significantly high germination (**Figures 7C,D**). To further analyze the effect of stress on *35S*::*OsSCP* transgenic plants, the root growth and fresh weight accumulation of 10-day-old wild-type and transgenic seedlings growing on 100 mM NaCl and 200 mM mannitol supplemented media were evaluated. The transgenic plants exhibited longer roots and accumulated significantly more fresh weight than wild-type plants at both salt and mannitol induced water-deficit stress condition (**Figure 8**).

#### Effect of Overexpression of *OsAMTR1* and *OsSCP* on Stress Responsive Genes

**FIGURE 9:**
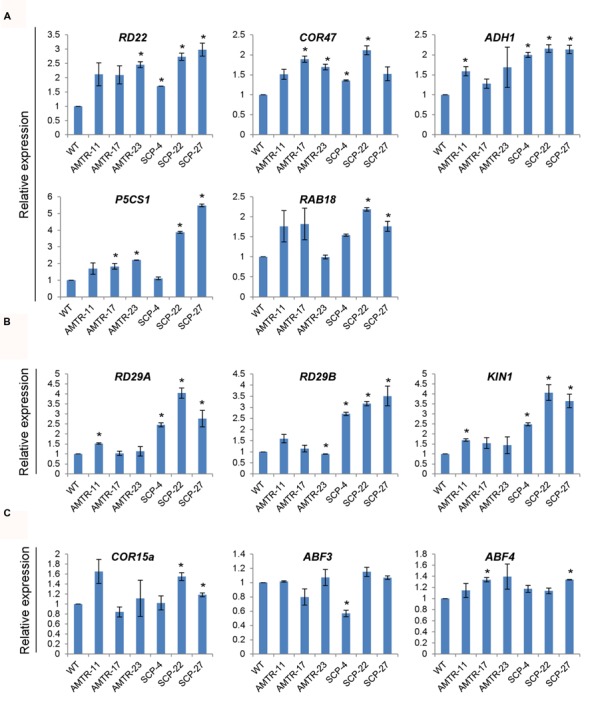
**Effect of overexpression of *OsAMTR1* and *OsSCP* in *Arabidopsis* on the expression of stress-responsive genes. qRT-PCR analysis of stress-responsive genes in 10-day-old seedlings of *35S*::*OsAMTR1* and *35S*::*OsSCP* transgenic plants and WT. (A)** Genes upregulated in both *35S*::*OsAMTR1* and *35S*::*OsSCP* plants, **(B)** genes upregulated in *35S*::*OsSCP* plants, **(C)** genes which have no change in expression in WT and either of transgenics. The graphs represent mean ± SE from three biological replicates. Asterisks represent significant difference with respect to wild-type as determined by *t*-test at *p*-value <0.05.

To analyze the effect of overexpression of *OsAMTR1* and *OsSCP* on stress signaling, the expression patterns of *Arabidopsis* known stress-responsive genes were analyzed in the *35S*::*OsAMTR1* and *35S*::*OsSCP* transgenic plants under normal growth conditions. A group of genes including *RD22*, *COR47*, *ADH1*, *P5CS1*, and *RAB18* were found to have increased expression in both the transgenics as compared to wild-type, which probably imparts higher stress tolerance to the transgenic plants (**Figure 9A**). In contrast, some genes like *RD29A*, *RD29B*, and *KIN1* are upregulated in *OsSCP* overexpressing plants but show similar expression levels as wild-type in *OsAMTR1* overexpressing plants, implying gene-specific effect on expression of *Arabidopsis* endogenous genes (**Figure 9B**). Likewise, some other genes, *ABF3*, *ABF4*, and *COR15a*, might not be involved in the stress responsive signaling in *35S*::*OsAMTR1* and *35S*::*OsSCP* transgenic plants as no change in expression of these genes was observed between wild-type and transgenic plants (**Figure 9C**).

## Discussion

### OsSAP1 Could Regulate Stress Responses by Interacting with Other Stress-Responsive Proteins

To understand the signaling mechanism of any protein, it is necessary to identify interacting proteins which are required for relay of signal. It is well-studied that homo- and hetero-complex formation of proteins can serve as a mechanism for their responses under stress (Nakashima et al., 2012; Nie et al., 2014; Zhu et al., 2014). For example, the homo- and hetero-dimerization between WRKY18, WRKY40, and WRKY60 has been shown to significantly affect their DNA binding activities (Xu et al., 2006). The self-association of SAPs can also have an effect on their stress responses. As SAPs are regulatory proteins, they can associate with many other proteins in the signaling pathway to regulate their activity under stress. With this idea in mind, the proteins which interact with OsSAP1 were identified using yeast two-hybrid library screening in rice. Two proteins, an aminotransferase (OsAMTR1) and a pathogenesis-related protein (OsSCP), were found to interact with OsSAP1 (**Figure 1**). Further, OsAMTR1 localizes in the form of irregular subcellular bodies in cytoplasm. These could be high molecular weight aggregates formed due to homo-heterodimers of OsAMTR1 or OsSAP1.

It is known that under salt and water-deficit stress, *OsSAP1* transcript starts accumulating within 15 min of stress (Mukhopadhyay et al., 2004), whereas OsSAP1 protein starts accumulating as early as 30 min of water-deficit stress and 3 h of salt treatment and keeps on increasing thereafter (Dansana et al., 2014). Similar to *OsSAP1*, *OsAMTR1*, and *OsSCP* were found to be responsive to salt and water-deficit stress condition at different levels. The expression profiles of all the genes were more or less similar under oxidative stress treatment and expression increased with time. This indicates high level of overlap in expression patterns between OsSAP1 and its interacting proteins. The temporal expression patterns can give an idea about the possible role of a gene during stress (Hasegawa et al., 2000; Singh et al., 2002). For instance, early induction of any gene under stress might be related to its function in early stress response. While, late expressing genes under stress can have a function under prolonged stress. Similarly, it has been suggested that co-expressing genes can be functionally related as it can be assumed that genes regulated by similar set of transcription factors can have similar expression patterns under a set of condition (van Noort et al., 2003; Allocco et al., 2004). Based on these hypotheses, OsSAP1 interacting proteins, *OsAMTR1* and *OsSCP*, having almost similar expression patterns as *OsSAP1*, were selected for further analyses.

### *OsAMTR1* Positively Regulates Salt and Water-Deficit Stress Response

Aminotransferases are a group of pyridoxal-5’-phosphate dependent enzymes that catalyze the reversible transfer of amino group from amino acids to oxo-acids and are involved in a number of metabolic activities (Liepman and Olsen, 2001). In plants, many aminotransferases have been found to play important roles in stress regulation, both biotic and abiotic. For instance, AlaATs from different plants have been suggested to play role in hypoxic stress (Ricoult et al., 2006; Rocha et al., 2010a,b). The homologs of AlaAT, alanine:glyoxylate aminotransferase (AGT) and glutamate:glyoxylate aminotransferase (GGAT) are involved in photorespiration and overexpression of these aminotransferases has shown higher resistance to many plant pathogens (Liepman and Olsen, 2001; Taler et al., 2004). Another aminotransferase ALD1 has been found to be a positive regulator of salicylic acid (SA)-mediated plant defense responses (Song et al., 2004; Nie et al., 2011). Besides this, a GABA transaminase (GABA-T) involved in GABA catabolism has been shown to play a role in plant salt stress tolerance (Renault et al., 2010).

Further, the YFP-OsAMTR1 fusion protein was found to be cytosolic. The plant alanine aminotransferases have been found to have different isoforms expressing in peroxisomes, mitochondria and cytosol and are involved in different cellular functions (Liepman and Olsen, 2003; Ricoult et al., 2006). In general, the peroxisomal forms have been identified to function in photorespiration while the mitochondrial forms are involved in hypoxic stress responses, but no specific function for cytosolic form has been described till date. All these evidences suggest that aminotransferases can have significant effect in the stress response of plants. *OsAMTR1* overexpressing plants showed enhanced tolerance under salt and water-deficit stress as evident from higher seed germination efficiency, root growth and biomass accumulation in transgenics as compared to wild-type. The observed positive regulation of known stress-responsive genes namely, *RD22*, *COR47*, *ADH1*, *P5CS1*, and *RAB18* in the transgenic plants might be responsible for imparting the stress tolerance to transgenic plants. In contrast, no deviation in the expression of *RD29A*, *RD29B*, *KIN1*, *ABF3*, *ABF4*, and *COR15a* was found in the transgenics than the wild-type plants suggesting these genes are not involved in the stress signaling pathway affected by *OsAMTR1*.

### Pathogenesis Related 1 (PR1) Protein Encoding Gene (*OsSCP*) Can Play a Role in Abiotic Stress Responses by Regulating Stress Responsive Genes

Pathogenesis-related (PR) proteins are group of proteins induced in plants in response to pathogen infection and are divided into 17 families (PR1 to PR17) based on the specific molecular function played by the family members (van Loon et al., 2006). In rice, *PR1* gene family comprises of 12 members and all of them have been found to be inducible by pathogen infection (Mitsuhara et al., 2008). Transcripts of two of the *PR1* genes, *OsPR1a* and *OsPR1b*, have been found to be inducible by JA, ethylene, SA, ABA, H_2_O_2_, protein phosphatase inhibitors and fungal infection (Agrawal et al., 2000). These inductions were dose-dependent and require *de novo* synthesis of some protein factor(s). In the present study, OsPR1a, named as OsSCP, has been identified to interact with OsSAP1 in yeast. The *OsSCP* transcript was found to be inducible by abiotic stress treatments such as water-deficit and MV induced oxidative stress. Earlier studies have also shown induction of *PR* genes in response to abiotic stress conditions such as salt stress, low temperature, and light (Snider et al., 2000; Zeier et al., 2004; Seo et al., 2008, 2010) suggesting important roles played by PR proteins in abiotic stress adaptation in addition to plant defense response against pathogens. Overexpression of *OsSCP* in *Arabidopsis* could enhance the tolerance of transgenic plants to salt and water-deficit stress as compared to wild-type plants. The *OsSCP* transgenic plants were also found to upregulate the expressions of *RD22*, *COR47*, *ADH1*, *P5CS1*, and *RAB18* but unlike in *35S*::*OsAMTR1* plants, expressions of *RD29A*, *RD29B*, and *KIN1* were also increased in *35S*::*SCP* plants, implying that in addition to some overlap, OsAMTR1 and OsSCP also have independent effects on stress related genes.

Plant PR1 proteins have been reported to exist in acidic and basic forms based on the isoelectric point (pI) of the protein (Van Loon and Van Strien, 1999; Mitsuhara et al., 2008). The acidic isoforms have been found to be secreted in extracellular space, whereas, the basic isoforms are localized in vacuoles (Dixon et al., 1991; Sessa et al., 1995). As expected, the subcellular localization of OsSCP, the acidic OsPR1a, has been predicted to be extracellular. However, OsSCP fused with YFP was found to be localized in the whole cell of onion epidermis and not in the extracellular space (Supplementary Figure S5). There could be a possibility that translocation of OsSCP to the extracellular space is condition dependent. So, the subcellular localization of OsSCP needs to be analyzed in detail by considering these possibilities.

### Possible Regulatory Mechanism of OsSAP1 under Abiotic Stress

The A20/AN1 containing proteins in animals possess E3 ubiquitin ligase activity and are involved in the regulation of inflammatory pathway via 26S proteasomal degradation pathway (Hishiya et al., 2006; Chang et al., 2011; Shembade and Harhaj, 2012). The plants SAPs have also been shown to possess E3 ligase activity (Kang et al., 2013; Sharma et al., 2015). Based on the recognition of different type of polyubiquitin chains, Lys48- or Lys63-linked, the ubiquitin binding protein can function in either degradation or regulating different cellular processes such as protein trafficking, DNA repair, protein activation or signal transduction (Pickart, 2001; Mural et al., 2013). The polyubiquitin recognition via A20 domain has been shown for *Arabidopsis* SAPs along with different affinities of AtSAP5 for Lys63- and Lys48-linked polyubiquitin chains (Choi et al., 2012). A similar activity has also been presumed for other SAPs, owing to high degree of homology in their A20 domain. This allows us to speculate that if OsSAP1 can add Lys63-linked polyubiquitin chains to its interacting proteins, then this interaction might affect activity of OsAMTR1 and OsSCP, which in turn influence the abiotic stress signaling. Also, OsSAP1 can regulate the biotic stress signaling pathway by regulating the expression of defense related genes including *PR* genes (Tyagi et al., 2014). Although, expression of *SCP*, also a PR gene, is not directly regulated by *OsSAP1* as its expression did not change in *OsSAP1* overexpression rice transgenics (Dansana et al., 2014), it could be speculated that OsSAP1-mediated alteration of OsSCP protein can converge the biotic and abiotic stress responses.

The signaling mechanisms involved in the stress response are complex and often interconnected. However, some elements are responsible for invoking specific stress responses that provide the specificity in the pathway (e.g., SOS pathway in salt stress response or ICE1 mediated cold stress response; Knight and Knight, 2001; Chinnusamy et al., 2004). It is evident that, except for *RD29A* and *RD29B*, all the stress responsive genes which were differentially expressed by the overexpression of *OsAMTR1* and *OsSCP* were not affected by the overexpression of *OsSAP1* (Giri, 2009). So, it could be speculated that OsSAP1 can regulate the stress responses by either modulating the expression of stress responsive genes or by affecting activity of its interacting proteins, which in turn can regulate the downstream stress responsive genes.

## Conclusion

The present study suggests that the OsAMTR1 and OsSCP interact with OsSAP1 and positively regulate the abiotic stress response in transgenic *Arabidopsis*. It may be stated that *OsSAP1* overexpression does not directly affect the expression of *OsAMTR1* and *OsSCP* in transgenic rice (Dansana et al., 2014). However, these proteins interact with OsSAP1 and SAPs have been shown to act as ubiquitin ligase, thereby affecting the interacting proteins which may be considered as possible mechanism of action for investigation in future. It may also be noted that the mechanism of action of AMTR1 and SCP may be variable or overlapping as discussed earlier.

## Author Contributions

KK performed all the experiments and wrote the manuscript. PD did BiFC experiment. AT conceived the idea. AT and JG designed the experiments, analyzed data and wrote manuscript. All authors approved final version of the manuscript.

## Conflict of Interest Statement

The authors declare that the research was conducted in the absence of any commercial or financial relationships that could be construed as a potential conflict of interest.
