# Supplementary material for: Rice Stress Associated Protein 1 (OsSAP1) Interacts with Aminotransferase (OsAMTR1) and Pathogenesis-Related 1a Protein (OsSCP) and Regulates Abiotic Stress Responses
Source: Front Plant Sci. 2016 Jul 19;7:1057. doi: 10.3389/fpls.2016.01057 (PMC4949214; doi:10.3389/fpls.2016.01057)
Supplement: Supplementary file 1 [file Image_1.PDF]

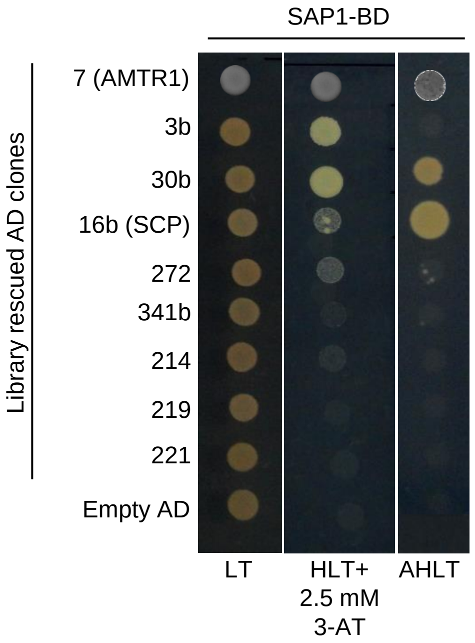

Figure S1. One-to-one yeast two-hybrid analysis using SAP1-BD and library rescued AD clones. The AD and BD constructs were co-transformed in yeast cells and plated on SD/-Leu/-Trp (LT), SD/-His/-Leu/-Trp+2.5 mM 3-AT (HLT+2.5 mM 3-AT) and SD/-Ade/-His/-Leu/-Trp (AHLT). Empty AD vector co-transformed with SAP1-BD served as a negative control.

```
1 GCCGGGAAGGAAGCCAACGCCGACGCCGACATGCAGCGCCTCGCCTCCTCCCGGAGGCTCCTCCAGGCGGCGCTCGCCCCTGTCCGTGCCGCCGTACGAC 100

101 GGGCCGCGGGCGGAGGAGATCTCCCGGAAGCGGGCCGAGTTCCTCAGCCCCTCCCTCTTCCACTTCTACGACCGCCCTTTGAACATAGTCGATGGGAAAA 200
M 1

201 TGCAGTACCTGTTCGACGAGGATGGCCGCCGTTACCTGGATGCTTTTGGTGGCATTGCAACCGTTTGTTGCGGGCACTGCCATCCGGATGTTGTTGAGGC 300
2 Q Y L F D E D G R R Y L D A F G G I A T V C C G H C H P D V V E A 34

301 GATGGTCAACCAAGCAAAGAGGATACAACACTCCACAGTTCTTTACCTGAATCATGCTATTGCAGATTTTGCCGAGGCATTGGCAGCCAAAATGCCTGGT 400
35 M V N Q A K R I Q H S T V L Y L N H A I A D F A E A L A A K M P G 67

401 GATCTGAAGGTTGTTTTCTTCACAAATTCTGGCACGGAGGCGAACGAGCTTGCACTGATGATTGCCCGGCTTTACACTGGTTGCCATGACATTATTTCGC 500
68 D L K V V F F T N S G T E A N E L A L M I A R L Y T G C H D I I S L 101

501 TCAGGAATGGATACCACGGGAATGCGGCTGGAACAATGGGTGCTACCGCTCAATGCAACTGGAAATTTAATGTTGTTCAGACGGGAGTGCACCATGCACT 600
102 R N G Y H G N A A G T M G A T A Q C N W K F N V V Q T G V H H A L 134

601 TAATCCAGACCCATATAGAGGTGCTTTCGGTTCTGATGGCGAAAAGTATGCCCGAGATGTTCAGGAAATCATTGATTTTGGAACTACAGGAAGGGTCGGT 700
135 N P D P Y R G A F G S D G E K Y A R D V Q E I I D F G T T G R V G 167

701 GGTTTCATTTCGGAAGCCATACAGGGAGTGGGTGGAATAGTGGAACTGGCACCAGGATACTTGCCTGCTGTGTACAATATGGTAAGGAAAGCTGGCGGCC 800
168 G F I S E A I Q G V G G I V E L A P G Y L P A V Y N M V R K A G G L 201

801 TCTGCATCGCCGACGAAGTTCAGGCGGGAGTCGCACGAACTGGAAACCACTTCTGGGGATTTGAATCACACGGCGTCATCCCAGACATAGTTACAATGGC 900
202 C I A D E V Q A G V A R T G N H F W G F E S H G V I P D I V T M A 234

901 AAAGGGTATAGGCAATGGCATACCGATCGGAGCAGTCGTCACAACCCCTGAGATCGCTCAGGTGTTAACCCGCAGGAGCTACTTCAACACCTTCGGTGGC 1000
235 K G I G N G I P I G A V V T T P E I A Q V L T R R S Y F N T F G G 267

1001 AACCCCGTCAGCACAGCCGGTGGCCATGCTGTTCTCAAGGTTCTGGAGAAGGAGAAGCTCCAGGAGAACGCATTTGTGGTTGGCTCCTACCTGAAGGAAC 1100
268 N P V S T A G G H A V L K V L E K E K L Q E N A F V V G S Y L K E R 301

1101 GGCTCAACAAGCTGAAAGAGAAGCATGACATCATTGGTGATGTTAGAGGGAAAGGCTTCCTTCTTGGGGTTGAGCTGGTGACCGATCGCCAGAAGAAGAC 1200
302 L N K L K E K H D I I G D V R G K G F L L G V E L V T D R Q K K T 334

1201 ACCGGCCAAAGTTGAGATCGGTCATGTCATGAACCACATGAAAGACATGGGTGTGCTGGTTGGGAAAGGTGGTTTCTACGGGAACGTGTTCAGAGTAACA 1300
335 P A K V E I G H V M N H M K D M G V L V G K G G F Y G N V F R V T 367

1301 CCTCCACTATGCTTCACCAAAGAGGACTCCGATTTCTTCATTGAGGCGATGGACATTTCACTATCGAAGCTGTGAGTCGGAGAGGGACTGGCAGGATCAA 1400
368 P P L C F T K E D S D F F I E A M D I S L S K L * 391

1401 CCTGTCCGCATGTGATCCGTATCCATAAATAATGTTGGGGATTGGTGAGATAGCACCTCGTTGTCTCGTAATCTGTCCTGCAAACCCACCATGCGCTGCT 1500

1501 CAACACATGGTCCAATAGGCGCTCCTCCGG 1530
```

Figure S2. Sequence analysis of *OsAMTR1*. The largest ORF obtained after 6-frame translation of *OsAMTR1* cDNA sequence has been shown. The red and blue coloured texts represent the 5’- and 3’-UTRs, respectively. The black coloured text is the coding sequence (CDS) of *OsAMTR1*. The shaded text represents the pyridoxal phosphate-dependent transferase domain in the OsAMTR1 protein.

```
  1 ACACAACCATACTAGCTAGTGATCTCTCGATCTCCATCATCTCTTCGTCTACTAACAAAGTTATAATATCAAATTAAGGTATATATATATATATATAGTA 100

101 GCTAGCTTCAATTAATGGCGAGTTCGTCGAGCAGGTTATCCTGCTGCTTGCTGGTGCTCGCGGCGGCGGCCATGGCGGCGACGGCGCAGAACTCGGCGCA 200
  1                M  A  S  S  S  S  R  L  S  C  C  L  L  V  L  A  A  A  A  M  A  A  T  A ▲Q  N  S  A  Q 29

201 GGACTTCGTGGACCCGCACAACGCGGCGAGGGCCGACGTCGGGGTGGGGCCGGTGAGCTGGGACGACACGGTGGCGGCGTACGCGGAGAGCTACGCGGCG 300
 30  D  F  V  D  P  H  N  A  A  R  A  D  V  G  V  G  P  V  S  W  D  D  T  V  A  A  Y  A  E  S  Y  A  A 62

301 CAGCGGCAGGGCGACTGCAAGCTGGAGCACTCGGACTCCGGCGGGAAGTACGGCGAGAACATCTTCTGGGGCTCCGCCGGCGGCGACTGGACGGCGGCGA 400
 63 Q  R  Q  G  D  C  K  L  E  H  S  D  S  G  G  K  Y  G  E  N  I  F  W  G  S  A  G  G  D  W  T  A  A  S 96

401 GCGCCGTGTCGGCGTGGGTGTCGGAGAAGCAGTGGTACGACCACGGCAGCAACAGCTGCTCGGCGCCGGAGGGGAGCTCGTGCGGGCACTACACGCAGGT 500
 97  A  V  S  A  W  V  S  E  K  Q  W  Y  D  H  G  S  N  S  C  S  A  P  E  G  S  S  C  G  H  Y  T  Q  V 129

501 GGTGTGGCGCGACTCGACGGCGATCGGCTGCGCCCGCGTCGTCTGCGACGGCGACCTCGGCGTCTTCATCACCTGCAACTACTCGCCGCCGGGCAACTTC 600
130  V  W  R  D  S  T  A  I  G  C  A  R  V  V  C  D  G  D  L  G  V  F  I  T  C  N  Y  S  P  P  G  N  F 162

601 GTCGGCCAATCTCCCTACTGATTAATTAATTAATTATATTATCACACTCACTAATTAATCATATCGTATGCTATGCTACGTGTTTATGCATGTATGGACA 700
163 V  G  Q  S  P  Y  * 168

701 TGTAGTGTCATATGGTATATCGTATATGTGTACGTATAATATACGTACGTATGCTGGTGAGAATAAATCCGATGAATAAATAAATAAAGCTGTACTGTCA 800

801 GCCGTATTTGCTTAGTGTATATACCGTGTATGCATTTACACTTTAGCTGTCCTTCGTGGTTACATGCATGGTCTGATGGTCATGTACATATATAGCTGGT 900

901 TTTTGCTGGTTAATTAATTTGTCCTTAATTTTGA 934
```

Figure S3. Sequence analysis of *OsSCP*. The largest ORF obtained after 6-frame translation of *OsSCP* cDNA sequence has been shown. The red and blue coloured texts represent the 5'- and 3'-UTRs, respectively. The black coloured text is the coding sequence (CDS) of *OsSCP*. The shaded text represents the CAP (Cysteine-rich secretory proteins, Antigen 5, Pathogenesis-related 1 proteins) domain in the OsSCP protein. The arrowhead denotes the cleavage site of the putative signal peptide.

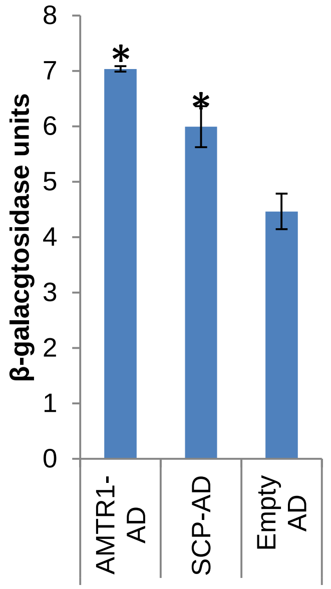

Fig S4. Quantification of beta-galactosidase activity in yeast cells co-transformed with SAP1ΔA20ΔAN1-BD with AMTR1-AD, SCP-AD and empty AD vector, separately. β-galactosidase activity was measured using ONPG as a substrate. Asterisks indicate significant difference from control at $p \leq 0.05$ by Student's *t*-test.

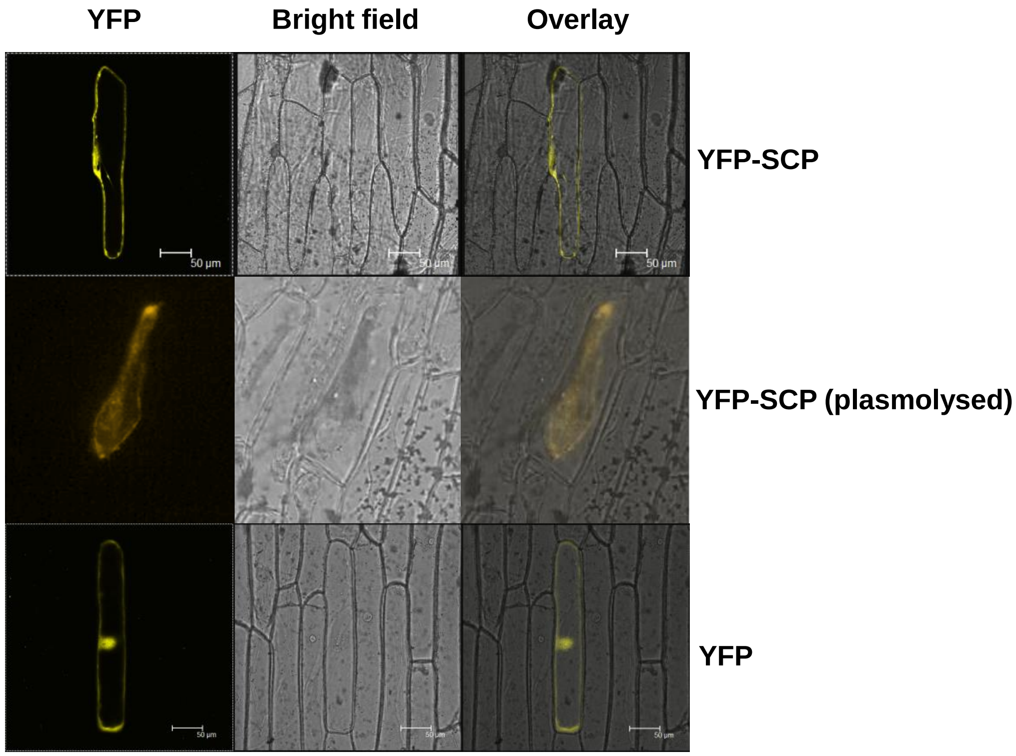

Figure S5. OsSCP has intracellular localization. The YFP fusion construct, YFP-SCP was transformed transiently in onion epidermal cells and was visualized by confocal microscope (upper panel). OsSCP-YFP localization in plasmolysed onion epidermal cells (middle panel). Empty vector (YFP) was used as a negative control (lower panel).

A

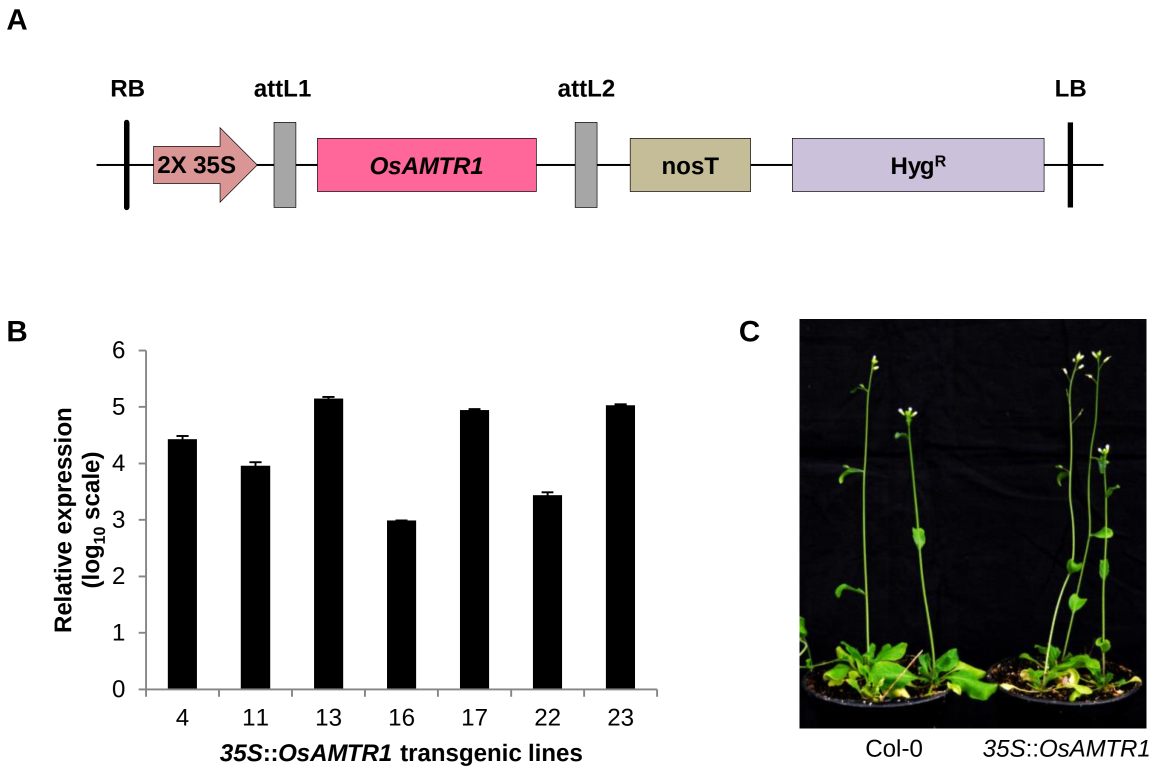

Figure S6. Generation of *Arabidopsis* transgenic plants overexpressing *OsAMTR1.* (A) Cloning of *OsAMTR1* under 2X *CaMV35S* promoter in binary vector pMDC32. (B) qRT-PCR analysis of transgenic *Arabidopsis* plants for *OsAMTR1* expression with respect to WT (Col-0) plants under unstressed condition. Relative expression levels have been shown in log_{10} scale. (C) Phenotype of five-week-old wild-type (Col-0) and *35S*::*OsAMTR1* plants.

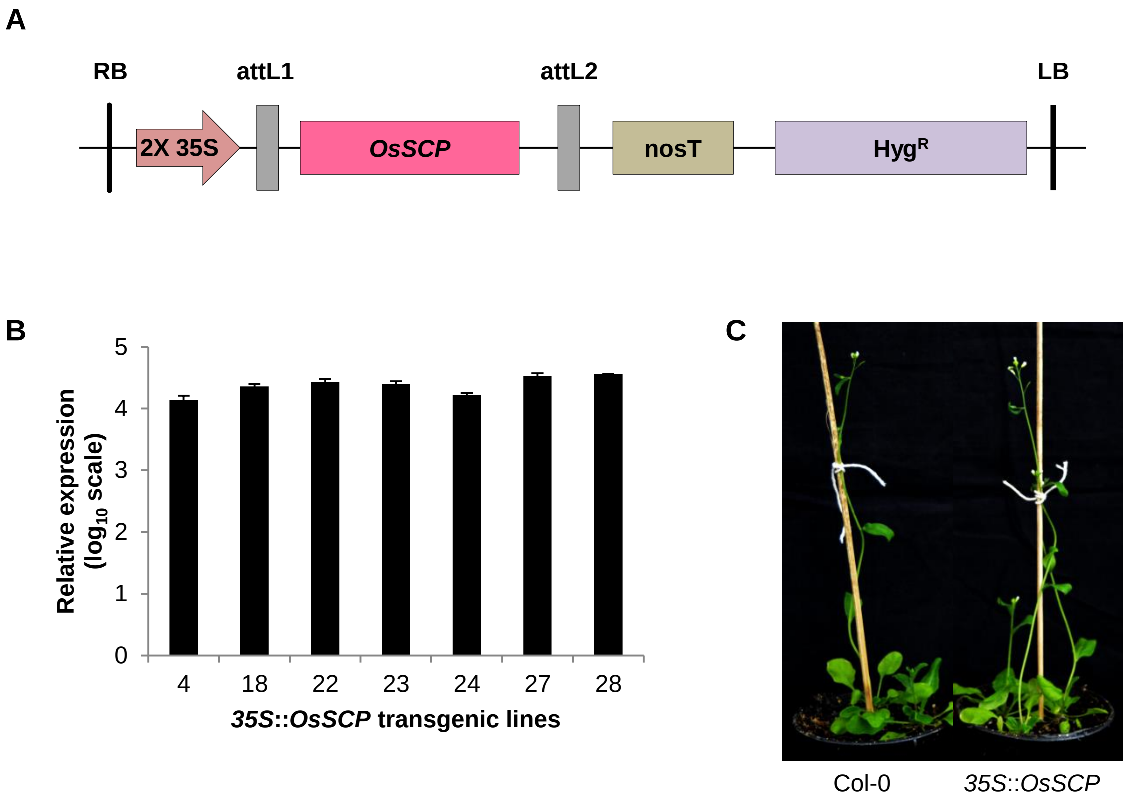

Figure S7. Generation of *Arabidopsis* transgenic plants overexpressing *OsSCP*. (A) Cloning of *OsSCP* under 2X *CaMV35S* promoter in binary vector pMDC32. (B) q-RT-PCR analysis of transgenic *Arabidopsis* plants for *OsSCP* transgene expression with respect to WT (Col-0) plants under unstressed condition. Relative expression levels have been shown in $\log_{10}$ scale.(C) Phenotype of five-week-old wild-type (Col-0) and *35S*::*OsSCP* plants.

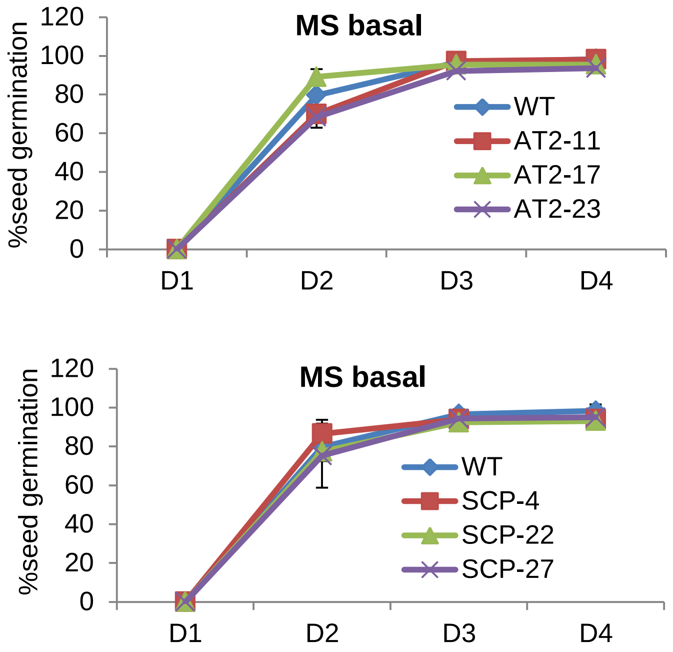

Fig S8. Germination efficiency of wild-type (WT) and *35S*::*OsAMTR1* and *35S*::*OsSCP* transgenic lines under unstressed condition. Wild-type (WT) and transgenic (line # AMTR-11, 17 and 23 of *35S*::*OsAMTR1*; SCP-4, 22 and 27 of *35S*::*OsSCP*) seeds were plated on MS basal medium and per cent germination was scored for 4 days after stratification. D1-D4 indicate days after transferring the plates to light in growth room.
