# Supplementary material for: Rice Stress Associated Protein 1 (OsSAP1) Interacts with Aminotransferase (OsAMTR1) and Pathogenesis-Related 1a Protein (OsSCP) and Regulates Abiotic Stress Responses
Source: Front Plant Sci. 2016 Jul 19;7:1057. doi: 10.3389/fpls.2016.01057 (PMC4949214; doi:10.3389/fpls.2016.01057)
Supplement: Supplementary file 2 [file Table_1.DOCX]

**Table S1. Primers used for gene cloning and generation of *Arabidopsis* transgenic plants**

| **Primer ID** | **Sequence (5’🡪3')** | **Purpose** |
| --- | --- | --- |
| SAP1_180_EcoRIF | ATAGAATTCGACAAGCAGCCGCCG | Cloning in pGBKT7 and pGADT7 vectors |
| SAP1_305_BamHIR | ATAGGATCCACCGCCGACGTCTTG |  |
| SAP1_13_EcoRI_F | ATAGAATTCGACAAGAAGGATCAGGAGC |  |
| SAP1_BamHI_R | ATAGGATCCTCAGAACCTAACGATCTTGG |  |
| SCP_EcoRI_F | ATAGAATTCATGGCGAGTTCGTCGA |  |
| SCP_BamHI_R | ATAGGATCCTCAGTAGGGAGATTGGCC |  |
| AMTR_EcoRI_F | ATAGAATTCATGCAGTACCTGTTCG |  |
| AMTR_BamHI_R | ATAGGATCCTCACAGCTTCGATAGTG |  |
| OsAMTR_UTR_F | ATCAACCACTTCACACGCGCGC |  |
| OsAMTR_UTR_R | TACGGATCACATGCGGACAGGTTG |  |
| AMTR_TOPO_F | CACCATGCAGTACCTGTTCGACGA | Cloning in pENTR-D/TOPO vector |
| AMTR_TOPO_Stop_R | TCACAGCTTCGATAGTGAAATGTCCATC |  |
| SCP_TOPO_F | CACCATGGCGAGTTCGTCGAGCA |  |
| SCP_TOPO_R | TCAGTAGGGAGATTGGCCGACG |  |
| T7_seq | TAATACGACTCACTATAGGGC | Sequencing |
| 3' _AD_seq | AGATGGTGCACGATGCACAG |  |
| 5'AD-LD amplimer | CTATTCGATGATGAAGATACCCCACCAAACCC |  |
| 3'AD-LD amplimer | GTGAACTTGCGGGGTTTTTCAGTATCTACGATT |  |
| 5' BD_amplimer | TCATCGGAAGAGAGTAGT |  |
| 3' BD_amplimer | AGAGTCACTTTAAAATTTGTAT |  |
| Hygro_F | TCTACACAGCCATGCGTCCAG |  |
| Hygro_R | GATGTAGGAGGGCGTGGATATG |  |

Note: Underlined sequence shows the restriction enzyme site

**Table S2. Sequence information of primers used for expression analysis**

| **Primer ID** | **Sequence (5’🡪3’)** | **Purpose** |
| --- | --- | --- |
| AMTR_RT_F | AGTGGAACTGGCACCAGGAT | Expression analysis in rice plants |
| AMTR_RT_R | CCGCCAGCTTTCCTTACCA |  |
| SCP_RT_F | TCTCCATCATCTCTTCGTCTACTAACAA |  |
| SCP_RT_R | GGATAACCTGCTCGACGAACTC |  |
| SAP1_RT_F | TTTTAATTGCAAACGGGAGGATA |  |
| SAP1_RT_R | TCGATTCTTTTTCCCTCAACCA |  |
| AT2_cds_RT_F | GAGGCGAACGAGCTTGCA | Expression analysis of transgene in *Arabidopsis* transgenic plants |
| AT2_cds_RT_R | AAATAATGTCATGGCAACCAGTGT |  |
| SCP_cds_RT_F | GGCGTACGCGGAGAGCTA |  |
| SCP_cds_RT_R | CAGAAGATGTTCTCGCCGTACTT |  |
| AtCOR47_F | TCGCTGTGTTTTGTGATCATTATCT | Expression analysis of stress responsive genes in *Arabidopsis* transgenic plants |
| AtCOR47_R | AAGGATCAAATGCAATCAACGA |  |
| AtP5CS1_F | AGTGTGTGTTTGTGTATTTGGTTGAG |  |
| AtP5CS1_R | GGAAACAAAAAAGCCCATCCT |  |
| AtRAB18_F | CCGCAGCATCTTGGATGTAA |  |
| AtRAB18_R | ATCCGAGATTCGAAACTAGTGAACA |  |
| AtRD29A_F | TGTTTGACGTCTTTGATGTATTATGG |  |
| AtRD29A_R | AGCCGAACAATTTATTAACCAAATG |  |
| AtRD29B_F | TTTTCGGATTCCGGTGGAA |  |
| AtRD29B_R | TCCCAGAATCTTGAACTCCCTTAC |  |
| AtCOR15a_F | AAACTCAGTTCGTCGTCGTTTCT |  |
| AtCOR15a_R | TCACCTTTAGCGGCGTAGATC |  |
| AtKIN1_F | AGCGGGAGGTGTTAACTTCGT |  |
| AtKIN1_R | TGACCCGAATCGCTACTTGTT |  |
| AtRD22_F | GCGATGATGACGACCGTCTA |  |
| AtRD22_R | TTAGCTCGCATCCCGTTCTC |  |
| AtABF4_F | TGTGCCCTGACCTTTGTTAGTTTA |  |
| AtABF4_R | GAAACCCTAACAACCAGAAAGCA |  |
| AtABF3_F | CCAACAAAGCGCCGAAGTTA |  |
| AtABF3_R | AACCTGCCTAAACTACGTACAAAGC |  |
| AtADHI_F | GGGAGAGAGTATTCGTTGCATCA |  |
| AtADHI_R | GTGAACATCATCTGCGAGAGAATG |  |
